# Insights into the Involvement of TRPA1 Channels in the Neuro-Inflammatory Machinery of Trigeminal Neuralgia

**DOI:** 10.3390/molecules30091884

**Published:** 2025-04-23

**Authors:** Chiara Demartini, Rosaria Greco, Anna Maria Zanaboni, Miriam Francavilla, Sara Facchetti, Cristina Nativi, Cristina Tassorelli

**Affiliations:** 1 Section of Translational Neurovascular Research, IRCCS Mondino Foundation, via Mondino 2, 27100 Pavia, Italy; chiara.demartini@mondino.it (C.D.); annamaria.zanaboni@unipv.it (A.M.Z.); miriam.francavilla@mondino.it (M.F.); sara.facchetti@mondino.it (S.F.); cristina.tassorelli@unipv.it (C.T.); 2 Department of Brain and Behavioral Sciences, University of Pavia, via Bassi 21, 27100 Pavia, Italy; 3 Dipartimento di Chimica “Ugo Schiff”, University of Florence, via della Lastruccia, 3-13, 50019 Sesto Fiorentino, Italy; cristina.nativi@unifi.it

**Keywords:** inflammation, TRPA1, glia, TLR4, TLR7

## Abstract

Antagonism of transient receptor potential ankyrin type-1 (TRPA1) channels counteracts the experimentally induced trigeminal neuralgia (TN) pain. TRPA1 channels activated/sensitized by inflammatory stimuli can modulate glial cell activity, a driving force for pathological pain. Additionally, the evidence of a link between TRPA1 and the inflammatory-related Toll-like receptors 4 (TLR4) and 7 (TLR7) highlights the potential of the TRPA1-blocking strategy to reduce pain and inflammation in TN. In this study, we aimed to further investigate the putative involvement of TRPA1 channels in the inflammatory pathways following the development of TN. We focused on the possible modulation of glial activity after TRPA1 blockade and the crosstalk of TRPA1 with TLR7 and TLR4. In a rat model of TN, based on chronic constriction injury of the infraorbital nerve, the impact of TRPA1 antagonism through ADM_12 treatment was assessed following the onset of mechanical allodynia (26 days post-surgery). The evaluation of central and peripheral inflammatory mediators (by rt-PCR and ELISA) and immunofluorescence staining of glial expression in the trigeminal nucleus caudalis was investigated using plasma samples and areas related to the trigeminal system (trigeminal ganglion and areas containing the trigeminal nucleus caudalis). Compared to sham-operated rats, the TN-like animals showed significant increases in the number of microglial and astroglial cells in the trigeminal nucleus caudalis, with higher and lower protein plasma levels of pro-inflammatory and anti-inflammatory cytokines, respectively. Additionally, in the trigeminal-related areas, TN-like animals showed significantly higher gene expression levels of TLR4, TLR7, miR-let-7b, and high-mobility group box-1. TRPA1 antagonism reverted all the observed alterations in TN-like rats in the trigeminal-related areas and plasma except microglial cell number in the trigeminal nucleus caudalis. The findings suggest that, in addition to their known involvement in the nociceptive pathway, TRPA1 channels may also play a direct or indirect role in pain-related inflammation, through the activation of TLR4- and TLR7-mediated pathways at the neuronal and glial levels.

## 1. Introduction

Trigeminal neuralgia (TN) is a chronic facial pain condition of multiple etiological origins, defined by the International Classification of Headache Disorders (ICHD-3) as a “disorder characterized by recurrent unilateral brief electric shock-like pains, abrupt in onset and termination, limited to the distribution of one or more divisions of the trigeminal nerve and triggered by innocuous stimuli” [1]. The mechanisms underlying TN are not yet fully understood; however, there is general agreement that the demyelination of trigeminal nerve fibers plays a significant role in its pathophysiology [2,3]. This process results in ectopic impulse generation and abnormal stimuli transmission, evolving into a state of chronic hyperexcitable and enhanced sensitivity [3]. In line with this, the first-line treatment for TN is the blockade of voltage-gated sodium channels (e.g., carbamazepine), which can reduce membrane hyperexcitation and ectopic nociceptive signaling [2]. However, disabling side effects and low drug tolerability are frequently reported, thus requiring surgical intervention [2], which is not always beneficial [4], highlighting the need for more effective therapeutic approaches.

Neuro-inflammation plays an important role in the molecular pathways that establish persistent orofacial pain in TN [5,6,7]. Indeed, in addition to the hyperactivation of trigeminal system-related neurons, glial cells and immune cells are also activated, generating a variety of inflammatory mediators that contribute to the enhancement of neuronal excitability [8]. It is thus suggested that modulating both neuron and glial abnormal activities would delineate the development of novel and more effective therapeutics. In this scenario, the transient receptor potential ankyrin type-1 (TRPA1) channels could represent a promising therapeutic target. These channels are known to be involved in pain perception and transmission, with a role in different types of pain ranging from nociception, acute pain, chronic pain, headache pain, and neuropathic pain [9]. Blockade or genetic ablation of TRPA1 channels was reported to have beneficial effects in counteracting trigeminal pain [10,11,12,13], including TN pain [14,15], although it is not clear whether these effects arise only from the inhibition of TRPA1 channels in nociceptive neurons or they also involve those found in non-neuronal cells. Notably, TRPA1 channels are present in both immune cells and glial cells [16,17,18,19]. Recent data have highlighted the involvement of TRPA1 channels in inflammatory-related mechanisms [12,13,20,21], thus suggesting the potential of the TRPA1-blocking strategy to reduce inflammatory pain. Evidence also suggests a connection between TRPA1 and certain Toll-like receptors (TLRs), which play a key role in the innate immune response [22]. TLR4 receptor activation was reported to upregulate TRPA1 in sensory neurons, thus contributing to pain [23]. A functional interaction, enhanced by the microRNA miRNA-let-7b, between TRPA1 and TLR7 receptors was reported [24], with implications in pain signaling [25]. In the present context, it is interesting to note that both TRL4 and TLR7 contribute to the development of neuropathic pain [26,27,28]. For example, pretreatment with high-mobility group box-1 (HMGB1), an endogenous ligand of TLR4, using a monoclonal antibody, prevented the development of trigeminal neuropathy in a murine model of TN [29].

In our previous study, we demonstrated the ability of TRPA1 antagonism to revert TN-like symptoms (i.e., mechanical allodynia); additionally, we showed that TRPA1 blockade downregulated the gene expression of pain neuropeptides and pro-inflammatory cytokines in trigeminal system-related areas [15]. In this study, we aimed to further investigate the potential role of TRPA1 channels in the inflammatory mechanisms involved in TN development. We specifically examined how glial activity might be influenced by blocking TRPA1, as well as the interaction between TRPA1, TLR7, and TLR4. To explore this further, we conducted additional experiments using the same samples from the animals involved in the earlier study [15], which established a rodent model of TN, based on chronic constriction injury (CCI) of the infraorbital nerve, alongside pharmacological modulation of TRPA1.

## 2. Results

### 2.1. Cytokine Modulation

**Figure 1:**
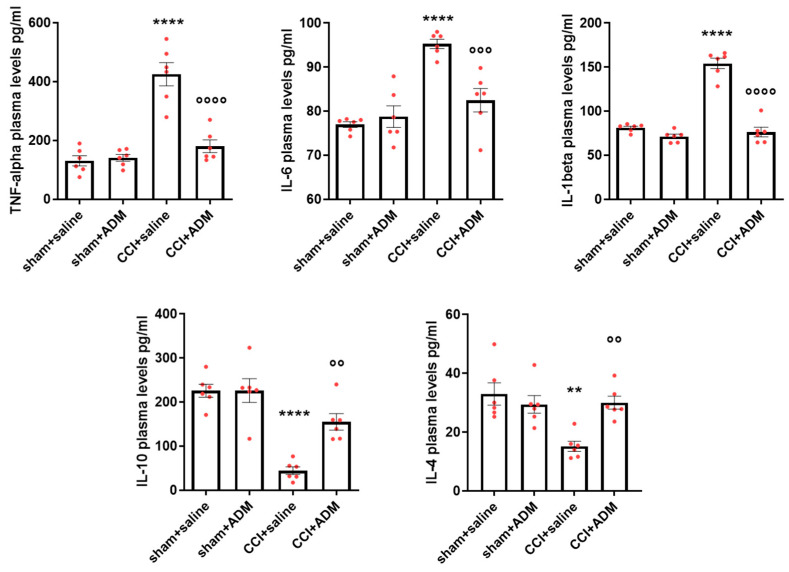
TNF-alpha, IL-6, IL-1beta, IL-10, and IL-4 protein plasma levels expressed as pg/mL. Data are expressed as mean ± SEM. One-way analysis of variance (ANOVA) followed by Tukey’s multiple comparison test, ** *p* < 0.01 and **** *p* < 0.0001 vs. sham + saline and sham + ADM, °° *p* < 0.01, °°° *p* < 0.001 and °°°° *p* < 0.0001 vs. CCI + saline. N = 6.

In CCI-operated animals, we found significantly altered protein plasma levels of pro- and anti-inflammatory cytokines compared to sham animals. Specifically, CCI animals displayed higher levels of tumor necrosis factor-alpha (TNF-alpha), interleukin (IL) 6, and IL-1beta, as well as lower levels of IL-10 and IL-4 (Figure 1). ADM_12 treatment in CCI-operated animals significantly reverted the CCI-induced changes in all cytokines, thus reducing the pro-inflammatory cytokines and increasing the anti-inflammatory ones (Figure 1). Additionally, we found consistent alterations in the respective cytokines’ gene expression analysis in the medulla in toto, cervical spinal cord (CSC), and trigeminal ganglia (TGs), both ipsi- and contralateral to the infraorbital nerve injury. Indeed, operated rats treated with AD_12 (CCI + ADM group) displayed a significant upregulation of *IL-10* and *IL-4* mRNA levels compared to CCI-operated animals (Supplementary Figure S1). In the previous study, we discovered in the same regions that ADM_12 may reduce the CCI-induced upregulation of *TNF-alpha*, *IL-6*, and *IL-1beta* [15].

ADM_12-injected sham rats showed no changes in cytokine gene or protein expression compared to the sham + saline group (Supplementary Figure S1 and Figure 1).

### 2.2. Glia Modulation

**Figure 2:**
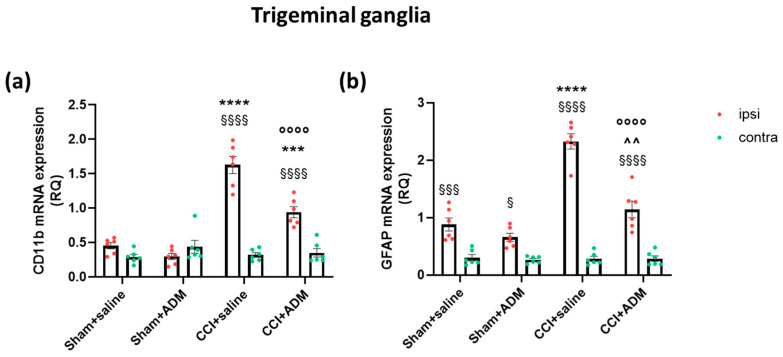
Gene expression in the trigeminal ganglia ipsi- and contralateral to the CCI/sham injury. mRNA levels, expressed as relative quantification (RQ), of (**a**) *CD11b* and (**b**) *GFAP*. Data are expressed as the mean ± SEM. Two-way ANOVA followed by Tukey’s multiple comparison test; *** *p* < 0.001 and **** *p* < 0.0001 vs. sham + saline and sham + ADM (ipsi); °°°° *p* < 0.0001 vs. CCI + saline (ipsi); ^^ *p* < 0.01 vs. sham + ADM (ipsi); § *p* < 0.05, §§§ *p* < 0.001 and §§§§ *p* < 0.0001 vs. contra. N = 6.

At the peripheral level, namely in the TGs, we investigated the gene expression levels of markers for resident macrophages and the satellite glial cell, cluster of differentiation molecule 11b (*CD11b*) and glial fibrillary acidic protein (*GFAP*), respectively. The CCI animals showed a strong upregulation of *CD11b* at the ipsilateral side compared to the contralateral side and to the sham groups (ipsilaterally). The ipsilateral *CD11b* increase of the operated animals was significantly counteracted by ADM_12 treatment (CCI + saline vs. CCI + ADM) (Figure 2a). *GFAP* gene expression levels were significantly upregulated at the ipsilateral side in all groups compared to the contralateral one (Figure 2b). Within the ipsilateral side, CCI animals showed significantly upregulated *GFAP* mRNA levels compared to the sham groups (Figure 2b). Treatment with ADM_12 in operated animals significantly reduced such upregulation (Figure 2b).

**Figure 3:**
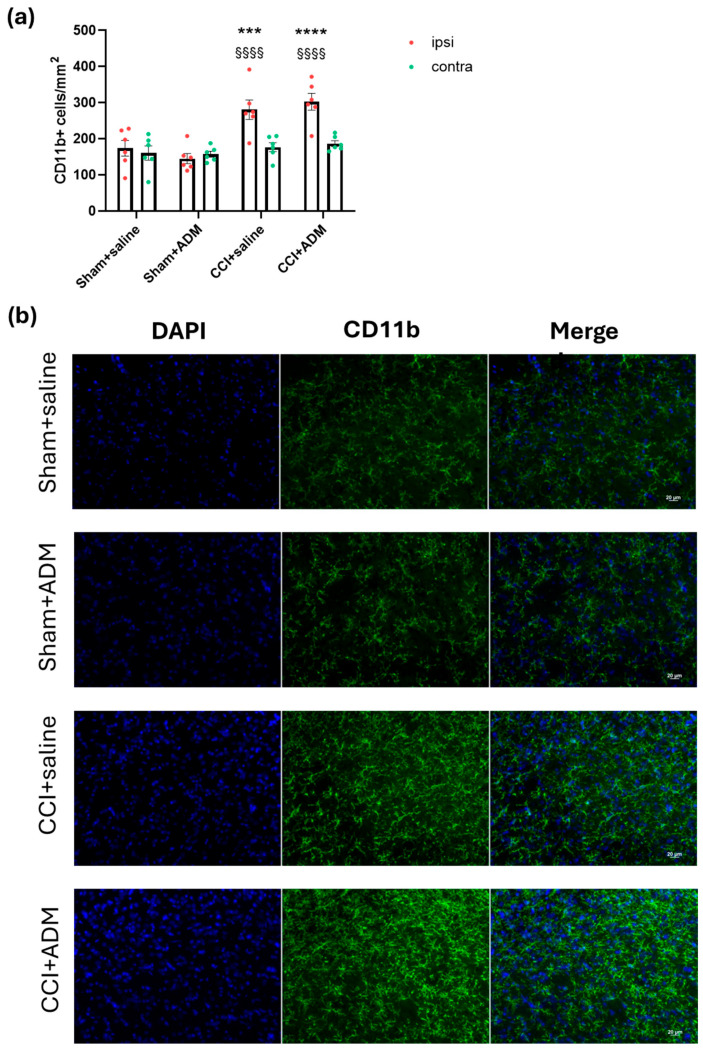
Microglial staining in TNC ipsi- and contralateral to CCI/sham injury. (**a**) Immunofluorescence analysis in the TNC area of CD11b-positive cells per area in mm^2^; (**b**) representative photomicrographs of microglial cells in the ipsilateral TNC (Scale bars: 20 µm. Blue: DAPI; green: CD11b). Data are expressed as the mean ± SEM. Two-way ANOVA followed by Tukey’s multiple comparison test; *** *p* < 0.001 and **** *p* < 0.0001 vs. sham + saline and sham + ADM (ipsi); §§§§ *p* < 0.0001 vs. contra. N = 6.

**Figure 4:**
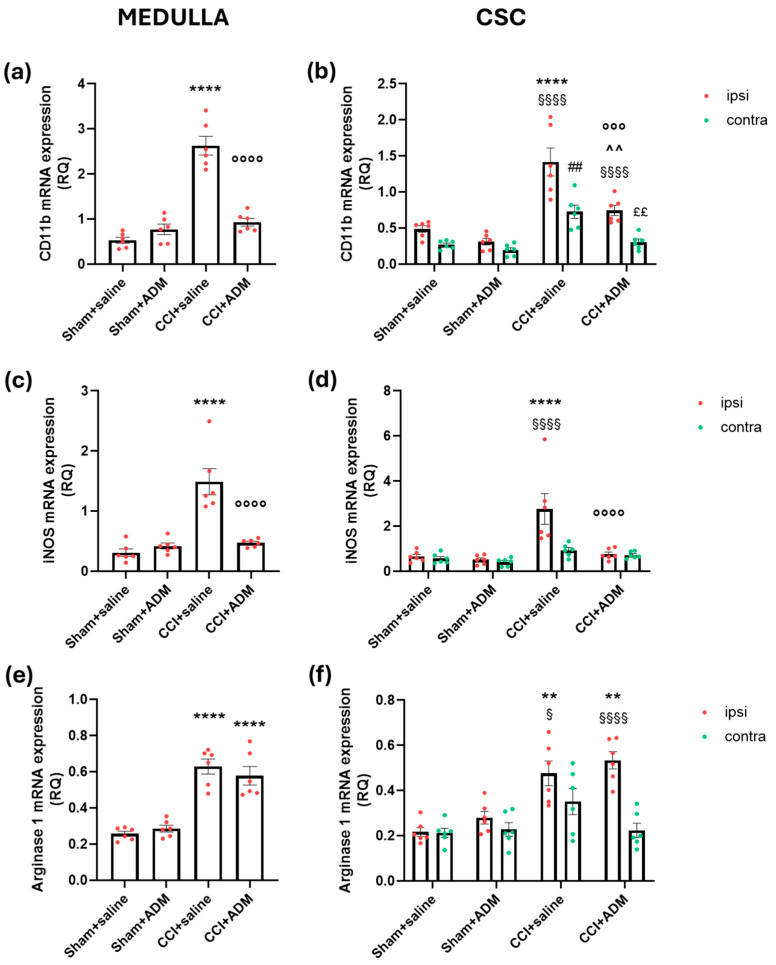
Microglia-related gene expression markers in the medulla in toto and cervical spinal cord (CSC) ipsi- and contralateral to CCI/sham injury. mRNA levels, expressed as relative quantification (RQ), of (**a**,**b**) *CD11b*, (**c**,**d**) *iNOS*, and (**e**,**f**) *Arginase 1*. Data are expressed as the mean ± SEM. One-way (for medulla) and two-way (for CSC) ANOVA followed by Tukey’s multiple comparison test; ** *p* < 0.01 and **** *p* < 0.0001 vs. sham + saline and sham + ADM (ipsi); °°° *p* < 0.001 and °°°° *p* < 0.0001 vs. CCI + saline (ipsi); ^^ *p* < 0.01 vs. sham + ADM (ipsi); § *p* < 0.05 and §§§§ *p* < 0.0001 vs. contra; ## *p* < 0.01 vs. sham + saline and sham + ADM (contra); ££ *p* < 0.01 vs. CCI + saline (contra). N = 6.

At the trigeminal nucleus caudalis (TNC) level of CCI rats (CCI + saline group), we found a strong increase in microglial cells in terms of the CD11b-positive cell number at both the ipsi- and contralateral sides compared to sham-operated animals (Figure 3). Accordingly, we observed higher levels of *CD11b* gene expression in the medulla and ipsilateral CSC in rats that had undergone CCI surgery (Figure 4a,b) compared to sham-operated animals. Furthermore, compared to the sham groups, the gene expression analysis of other microglial markers, namely inducible nitric oxide synthase (*iNOS*) and *Arginase 1*, revealed a significant upregulation in CCI + saline rats (Figure 4c–f). ADM_12 treatment of the operated animals was unable to modulate the number of microglial cells in TNC (Figure 3), while it downregulated *CD11b* and *iNOS* mRNA levels compared to the CCI + saline group (Figure 4a–d); by contrast, no effects were reported on *Arginase 1* (Figure 4e,f).

**Figure 5:**
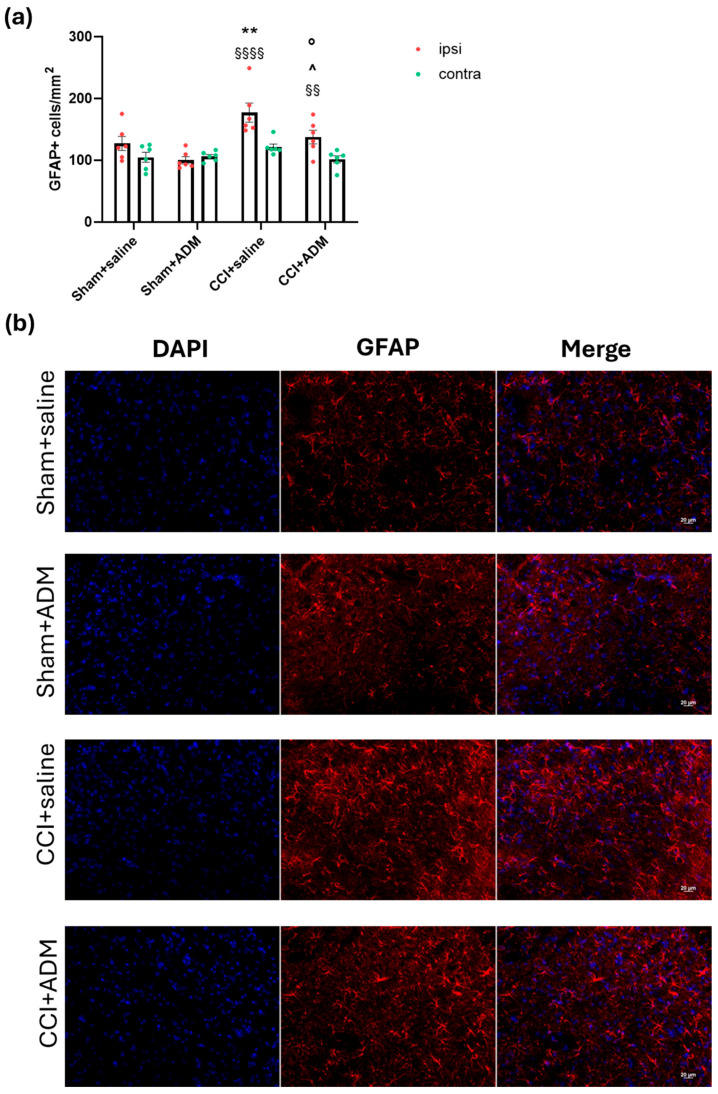
Astroglial staining in TNC ipsi- and contralateral to CCI/sham injury. (**a**) Immunofluorescence analysis in the TNC area of GFAP-positive cells per area in mm^2^; (**b**) representative photomicrographs of astroglial cells in the ipsilateral TNC (Scale bars: 20 µm. Blue: DAPI; red: GFAP). Data are expressed as the mean ± SEM. Two-way ANOVA followed by Tukey’s multiple comparison test; ** *p* < 0.01 vs. sham + saline and sham + ADM (ipsi); ° *p* < 0.05 vs. CCI + saline (ipsi); ^ *p* < 0.05 vs. sham + ADM (ipsi); §§ *p* < 0.01 and §§§§ *p* < 0.0001 vs. contra. N = 6.

**Figure 6:**
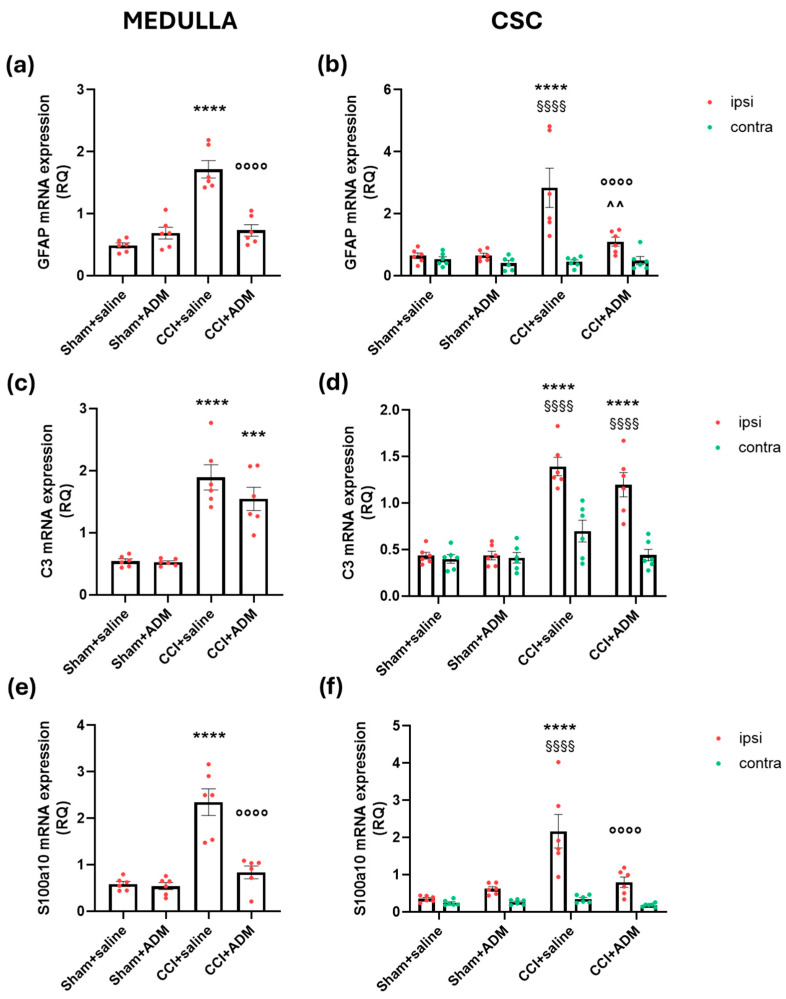
Astroglia-related gene expression markers in the medulla in toto and cervical spinal cord (CSC) ipsi- and contralateral to CCI/sham injury. mRNA levels, expressed as relative quantification (RQ), of (**a**,**b**) *GFAP*, (**c**,**d**) *C3*, and (**e**,**f**) *S100a10*. Data are expressed as the mean ± SEM. One-way (for medulla) and two-way (for CSC) ANOVA followed by Tukey’s multiple comparison test; *** *p* < 0.001 and **** *p* < 0.0001 vs. sham + saline and sham + ADM (ipsi); °°°° *p* < 0.0001 vs. CCI + saline (ipsi); ^^ *p* < 0.01 vs. sham + ADM (ipsi); §§§§ *p* < 0.0001 vs. contra. N = 6.

Similarly to microglia, the CCI + saline group showed a more marked increase in astroglial cells in the ipsilateral TNC, evidenced by an increased number of GFAP-positive cells (Figure 5) and upregulation of the *GFAP* gene in the medulla and ipsilateral CSC (Figure 6a,b) when compared with the sham groups. CCI rats also had upregulated mRNA levels in the medulla and ipsilateral CSC of other astroglial markers, namely Complement 3 (*C3*) and S100 Calcium-Binding Protein A10 (*S100a10*), compared to sham animals (Figure 6c–f). A single injection of the TRPA1 antagonist reduced GFAP-positive cells in the ipsilateral TNC (Figure 5); moreover, ADM_12 was able to downregulate the CCI-induced increase in *GFAP* and *S100a10* mRNA levels (Figure 6a,b,e,f), but not those of *C3* in the medulla and ipsilateral CSC (Figure 6c,d).

TRPA1 was expressed only by astroglial cells and not by microglial cells after double immunostaining with GAFP and CD11b in the TNC (Supplementary Figure S2).

The sham + saline and sham + ADM groups did not differ in any of the data shown in Figure 2, Figure 3, Figure 4, Figure 5 and Figure 6.

### 2.3. Changes in TLR-Related Pathways

**Figure 7:**
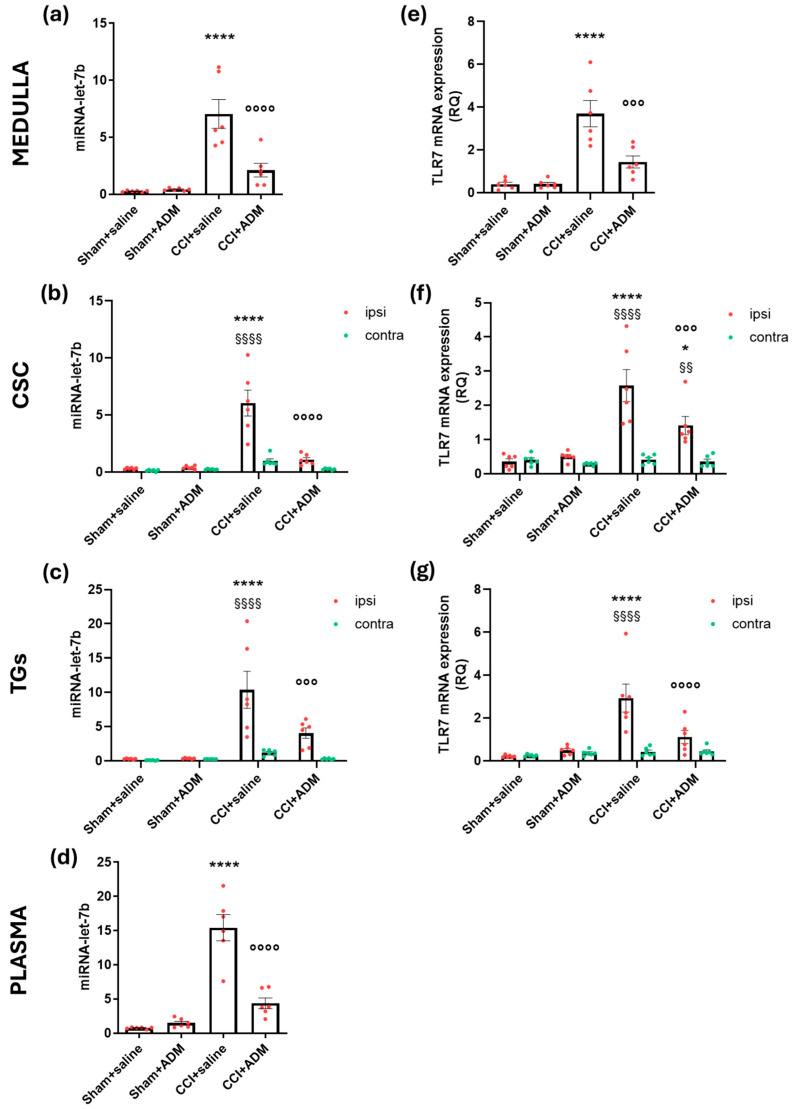
Gene expression of miR-let-7b and *TLR7* in the medulla in toto, cervical spinal cord (CSC) and trigeminal ganglia (TGs) ipsi- and contralateral to CCI/sham injury and in plasma. Gene expression, expressed as relative quantification (RQ), of (**a**–**d**) miR-let-7b and (**e**–**g**) *TLR7*. Data are expressed as the mean ± SEM. One-way (for medulla and plasma) and two-way (for CSC and TGs) ANOVA followed by Tukey’s multiple comparison test; * *p* < 0.05 and **** *p* < 0.0001 vs. sham + saline and sham + ADM (ipsi); °°° *p* < 0.001 and °°°° *p* < 0.0001 vs. CCI + saline (ipsi); §§ *p* < 0.01 and §§§§ *p* < 0.0001 vs. contra. N = 6.

**Figure 8:**
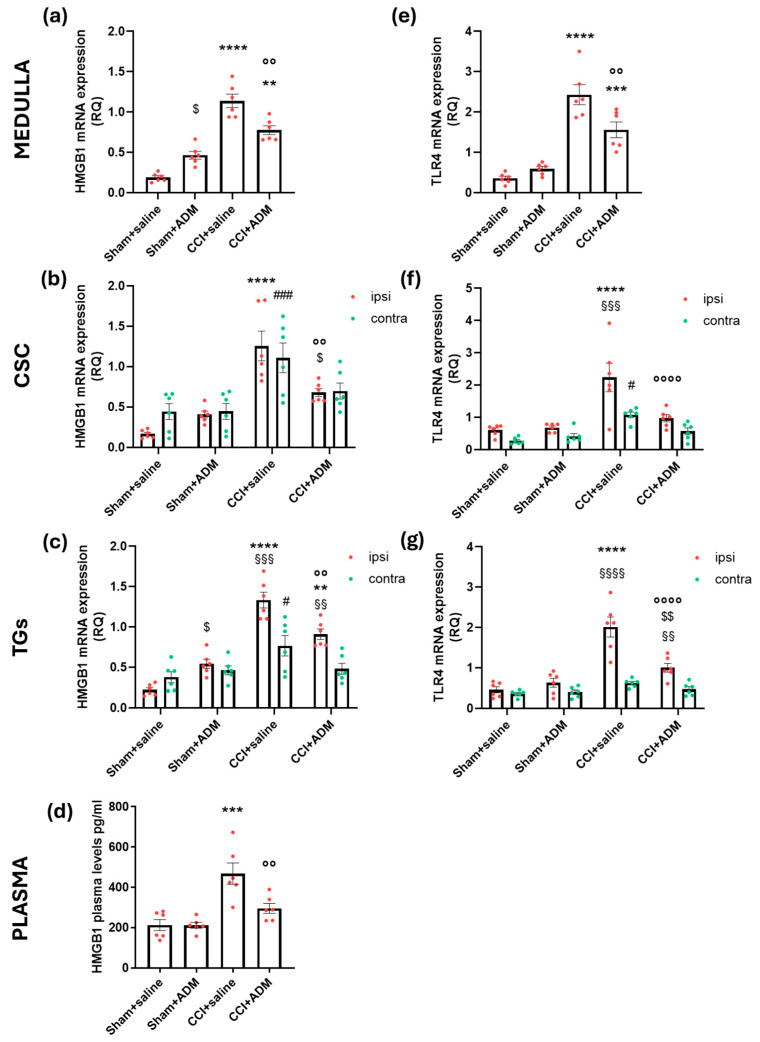
Gene expression of *HMGB1* and *TLR4* in the medulla in toto, cervical spinal cord (CSC) and trigeminal ganglia (TGs) ipsi- and contralateral to CCI/sham injury and HMGB1 protein in plasma. mRNA levels, expressed as relative quantification (RQ), of (**a**–**c**) *HMGB1* and (**e**–**g**) *TLR4*; (**d**) protein plasma levels expressed as pg/mL of HMGB1. Data are expressed as the mean ± SEM. One-way (for medulla and plasma) and two-way (for CSC and TGs) ANOVA followed by Tukey’s multiple comparison test; ** *p* < 0.01, *** *p* < 0.001 and **** *p* < 0.0001 vs. sham + saline and sham + ADM (ipsi); °° *p* < 0.01 and °°°° *p* < 0.0001 vs. CCI + saline (ipsi); $ *p* < 0.05 and $$ *p* < 0.01 vs. sham + saline (ipsi); # *p* < 0.05 and ### *p* < 0.001 vs. sham + saline and sham + ADM (contra); §§ *p* < 0.01, §§§ *p* < 0.001 and §§§§ *p* < 0.0001 vs. contra. N = 6.

Gene expression analysis revealed augmented levels of miR-let-7b (whose expression is higher even at the plasma level) (Figure 7a–d), *TLR7* (Figure 7e–g), *TLR4* (Figure 8e–g), and *HMGB1* (Figure 8a–c) in the central areas (medulla and CSC), as well as the peripheral one (TGs) of the CCI animals compared to sham-operated rats; additionally operated animals showed higher HMGB1 protein plasma levels (Figure 8d) compared to sham groups. ADM_12 treatment in CCI-operated animals significantly reduced the CCI-induced changes (CCI + saline vs. CCI + ADM groups) (Figure 7 and Figure 8).

Remarkably, compared to the sham + saline group, the administration of ADM_12 to sham-operated animals raised the levels of *HMGB1* gene expression in the medulla and the ipsilateral TG (Figure 8a,c). There were no reported differences between the sham + saline and sham + ADM groups regarding miR-let-7b, *TLR7*, or *TLR4*.

## 3. Discussion

TN is an orofacial pain that is difficult to diagnose and treat, with many unknown mechanisms underlying its pathophysiology. Among the variety of pain mediators, TRPA1 channels seem to play a key role in the development of neuropathic pain. Of interest, it was reported that genetic variants of the TRPA1 gene, among others, were found in familial TN subjects [30], thus highlighting their involvement in neuropathic pain. Indeed, different models of neuropathic pain, including the model based on the CCI of the infraorbital nerve, showed that *TRPA1* mRNA [15] and protein [31] are upregulated; additionally, a blockade or genetic ablation of these channels reduces pain behavior together with other pain markers [14,15].

Previously, we found that animals treated with ADM_12 could reverse trigeminal mechanical allodynia and downregulate *TRPA1* mRNA along with the neuropeptide substance P and calcitonin gene-related peptide [15]. Here, we investigated how TRPA1 antagonism affects the inflammatory processes underlying TN.

### 3.1. TRPA1 as an Inflammatory Modulator Through the Crosstalk with the TLR-Related Pathways

This study found that a single administration of ADM_12 reversed cytokine expression changes in rats subjected to the CCI of the infraorbital nerve. In addition to the previously reported reduction in pro-inflammatory cytokine gene expression [15], we found a significant upregulation of anti-inflammatory cytokines in trigeminal-related areas after TRPA1 antagonism. Notably, these alterations in mRNA expression at the nervous system tissue level were consistent with altered peripheral protein plasma levels of pro- (TNF-alpha, IL-1beta, IL-6) and anti-inflammatory (IL-4, IL-10) cytokines. These data are in line with previous studies, achieved in different models, in which the inhibition of TRPA1 channels resulted in anti-inflammatory effects with reduced pro-inflammatory cytokines and an increase in the anti-inflammatory ones [20].

The changes observed in the present study may be related to the TRPA1 localized in glial cells; indeed, TRPA1 was identified in satellite glial cells [16] and Schwann cells [32] at the peripheral level, as well as on oligodendrocytes [33] and astrocytes centrally [18,34], a finding also confirmed in the present study. The role of TRPA1 in glial cells is still not fully understood, but it seems that they are involved in different types of pathways. For instance, TRPA1 in Schwann cells may contribute to oxidative stress generation [32]; on the other hand, in satellite glial cells and astrocytes, TRPA1 channels are responsible for the regulation of basal calcium levels [16,18], especially in pathological conditions [35].

In the current study, we observed a downregulation of *CD11b* and *GFAP* mRNA at the TG level following TRPA1 antagonism. Together with the reduction in gene expression of pro-inflammatory cytokines and an increase in the anti-inflammatory ones [15], this finding suggests a generalized reduction in satellite glial cell activity as well as a possible decrease in infiltrating/resident macrophages. TRPA1 channels were found to be expressed and functionally active in satellite glial cells and to be increased after inflammatory and neuropathic pain models [16]. Therefore, we can hypothesize that their blockade, with consequent reduction in calcium influx, may have induced changes in basal cell activity toward an anti-inflammatory profile [20]. Additionally, TRPA1 channels may also be expressed in macrophages [36], which were recognized as the main source of *IL-1beta* increase in ganglia after nerve injury [37]. Furthermore, the concomitant blockade of TRPA1 channels located on the soma of TG neurons may result in a reduced release of transcellular communication molecules among neurons, macrophages, and satellite glial cells, thus regulating neuronal hyperexcitability [38].

Similarly, at the central level, namely in the medulla and CSC, we found a downregulation of *CD11b* and *GFAP* mRNA in the CCI animals treated with ADM_12. This is partially reflected by a reduction in the immunoreactivity of GFAP at the TNC level but not of CD11b. Such an apparent discrepancy could be the result of the dynamic microglial changes [39] that resulted in an unchanged number of cells. Even if this is not related to the sole TNC nucleus, we found that in the medulla and CSC areas, the gene expression of the microglial markers, *iNOS* and *Arginase1*, and the astroglial ones, *C3* and *S100a10*, were significantly increased in TN-like operated animals compared to sham rats. The iNOS and C3 markers are generally linked to pro-inflammatory profiles of microglial and astroglial cells, respectively [40]; Arginase 1 and S100a10 are associated with the anti-inflammatory states of microglia and astroglia, respectively [40]. It is crucial to recognize that a single marker (and a single technique) cannot accurately establish the status and function of the relevant glial cells [39,41]. Nonetheless, these data support the multifaced effects of glial cells after nerve injury [42,43].

In the presence of the TRPA1 antagonist in neuropathic animals, only *iNOS* and *S100a10* were significantly downregulated. These puzzling results may be related to a direct effect on astrocytes and an indirect effect on microglial cells, which warrants further studies. As confirmed in this study, TRPA1 channels are expressed in astrocytes but not in microglial cells. Notably, Lee and co-workers showed a significant increase in TRPA1 expression in the fine processes of astrocytes in the TNC of rats subjected to temporomandibular joint inflammation [44]. Consistently, we previously reported upregulated levels of *TRPA1* mRNA in the areas containing the TNC after induction of experimental trigeminal neuralgia in rats [15]. It can be hypothesized that blocking TRPA1 channels in astrocytes facilitated cellular pathways directed to the astroglia–microglia crosstalk, thus indirectly modulating microglial activity. *TRPA1* knockdown was shown in mice to inhibit microglia activation in the lumbar spinal cord generated by kinin B1 receptor activation [45]. Thus, similarly to earlier studies [12,46], TRPA1 channels may influence not only astrocytes but also microglia activity even if they are not expressed in those types of cells.

One of the possible ways through which TRPA1 channels can modulate the inflammatory machinery is a putative crosstalk with the TLR4- and TLR7-related pathways.

TLR4 and TLR7 receptors are known to be mainly involved in immune and inflammatory responses [22]; however, substantial evidence also supports their role in pain modulation. Indeed, they contribute to the development and maintenance of central sensitization by generating inflammatory mediators [47]. In agreement with other studies on neuropathic pain models [27,48,49,50], here, we found a significant increase in *TLR4* and *TLR7* gene expression in trigeminal-related areas of TN-like animals, thus supporting their impact on the development of neuropathic pain [26,27]. Additionally, in the same areas, and also in plasma, we found upregulated levels of the TLR7 endogenous agonist miR-let-7b and the TLR4 ligand HMGB1 in CCI-operated rats. These latter findings are consistent with literature data, indicating a role for both miR-let-7b and HMGB1 in neuropathic pain [25,29]. Interestingly, following TRPA1 blockade with ADM_12 in TN-like animals, all the observed changes were reverted.

TLR4 and TLR7 receptors can modulate TRPA1 channels. Indeed, TLR4 activation was reported to upregulate TRPA1 in sensory neurons contributing to pain [23], and TLR7 was reported in vitro (in dorsal root ganglion neurons) to functionally interact with TRPA1 [24]. Such interaction seems to be enhanced by the microRNA miRNA-let-7b, an endogenous agonist of TLR7, which in turn activates TRPA1 channels [24]. Moreover, Chen and collaborators [25] recently discovered that miR-let-7b is implicated in pain signaling in vivo through both neuronal and glial signaling with mechanisms that involve TLR7 receptors and TRPA1 channels. It is interesting to note that miRNA-let-7b was found to be altered in the blood of patients with complex regional pain syndrome [51] as well as in dorsal root ganglion from rats subjected to spinal nerve ligation [52]. The TLR7 activation achieved by miR-let-7b could also be facilitated by HMGB1, which is able to form heterodimeric complexes with miR-let-7b [53]. HMGB1 is an endogenous ligand of TLR4 receptors, promoting inflammation via nuclear factor kappa B (NF-kB) activation [54], which plays a key role in the neuroimmune crosstalk [55]. Notably, the HMGB1/TLR4/NF-kB axis was reported to be deeply involved in the inflammatory response in neuropathic pain conditions [56,57].

### 3.2. Overview

**Figure 9:**
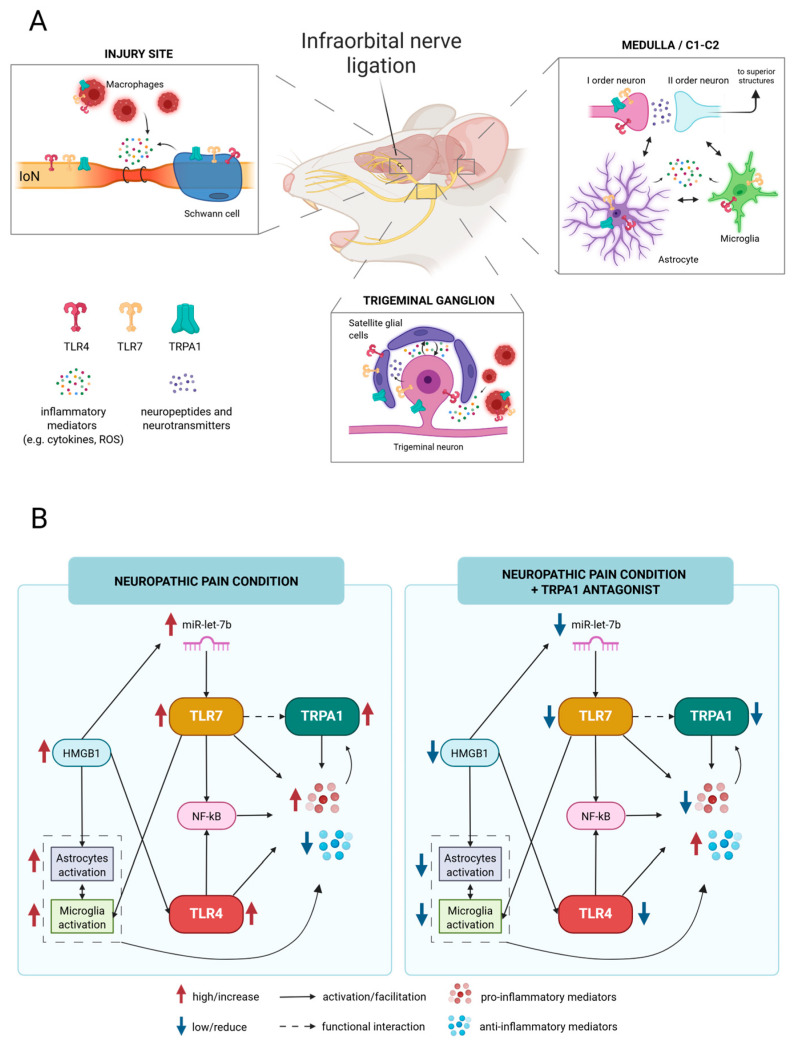
Schematic illustration of the proposed inflammatory mechanisms involving TRPA1 channels in TN: (**A**) The primary structures and cells implicated in the generation of trigeminal neuropathic pain, including those expressing TRPA1 channels as well as TLR4 and TLR7 receptors, located at the injury site, in the trigeminal ganglion and within the medulla/C1-C2 region. (**B**) The potential mechanisms and interactions associated with TRPA1 channels.

Following nerve damage, injury discharges are conveyed through the TG to the TNC in the brainstem and then to the higher CNS structures. Along the pathway, many non-neuronal cells, producing a variety of inflammatory mediators, are involved and actively participate in the development and maintenance of neuropathic pain. Figure 9A shows the main structures and cells that are involved in the trigeminal neuropathic pain generation, together with the localization of the TRPA1 channels, as well as TLR4 and TRL7 receptors. Specifically, TLRs and TRPA1 are expressed in primary trigeminal neurons, Schwann cells, macrophages, satellite glial cells, and astrocytes. However, TRPA1 is notably absent in microglia. Figure 9B illustrates the potential mechanisms and interactions involving TRPA1 channels.

In this study, we found high expression levels of miR-let-7b, *TLR7*, *TLR4*, and *HMGB1*, contributing to the marked increase in glial cell activation with the consequent release of pro-inflammatory mediators. Indeed, HMGB1 was reported to enhance neuropathic pain by mediating astrocyte activation and the TLR4/NF-kB signaling pathway [58]. In addition, HMGB1 may facilitate the action of miR-let-7b, which can agonize TLR7. The latter contributes to pain generation via microglia activation [25], NF-kB-mediated pathways [27], and activation (with functional interaction) of TRPA1 channels [24,25], whose levels are elevated in pain conditions [15,31]. Neuro-inflammatory mediators (e.g., cytokines, reactive oxygen species), also mediated by the TLR4 and TLR7 signaling pathways, can stimulate and increase the activity (and levels) of TRPA1 channels [20,59]. For instance, TNF-alpha was reported to facilitate the plasma membrane insertion of TRPA1 in rat sensory neurons, thus amplifying their effects [60]. Moreover, the activation of the NF-κB signaling pathway, promoted by pro-inflammatory cytokines, was reported to induce *TRPA1* gene expression [61]. In turn, the increased activity and levels of TRPA1 channels lead to a modulation of glial cells and to a further release of inflammatory mediators [20].

It is plausible to assume that, as a result of the nerve injury, a self-feeding mechanism was established in which inflammatory stimuli prompted the activation and increased expression of TRPA1 channels, either at the neuronal or glial level, where they may have contributed to the increased release of pro-nociceptive neuropeptides [62] and pro-inflammatory cytokines [34], respectively. Blockade of TRPA1 channel may have influenced the crosstalk between glial cells and neurons [40] and disrupted, with feedback mechanisms, the miR-let-7b/TLR7/TRPA1 and HMGB1/TLR4/NF-kB axes, thereby reducing the downstream inflammatory molecules. All these events, occurring across multiple cellular substrates, collectively lead to a decrease in inflammatory pain, ultimately causing the attenuation of pain-related behaviors [15], suggesting that the combination of TRPA1 antagonists with inhibitors of TLR signaling could amplify their therapeutic effects.

### 3.3. Limitations of the Study

This study contributes small but critical pieces to our knowledge of the intricate processes that underlie TN pain, but it is essential to recognize some limitations.

First, there is an inherent restriction to this study: additional functional data, to further corroborate our findings, are not included because the same animal samples used here belong to the previously published study (thus avoiding animal waste). Therefore, additional research will address this point. Second, whereas TRPA1 channels appear to play a crucial role in pain and inflammation processes, their precise mechanism of action is still unclear. The findings also raise intriguing questions about the cell-specific functions of TRPA1 channels. It is worth noting that the markers used in this study may not have fully captured the potential differences or changes that occurred. Indeed, glial morphology and function are highly dynamic and complex [39,41]. Evaluating additional markers and employing diverse techniques will be essential to provide confidence in the findings presented. Furthermore, future studies should prioritize investigating the activity and functionality of TRPA1 channels, particularly their potential functional interplay with TLRs. Finally, given the intriguing effect that ADM_12 may exert on the transient receptor potential vanilloid-1 channels [10,15], more research specifically aimed at ruling out the involvement of these channels (such as particular pharmacological tools) in this context will be required in the future to completely define the role of TRPA1 channels.

## 4. Materials and Methods

### 4.1. Experimental Plan

To adhere to the 3R principles, i.e., reducing/avoiding animal use if not necessary, all the experiments performed in this study were conducted using samples obtained from the cohort of animals described in our previous study [15].

Based on our previous data on the gene expression levels of *IL-1beta* in the medulla [15], we conducted a post hoc analysis to evaluate the achieved power, obtaining a power of 0.97. Thus, to evaluate the inflammatory-related pathways in the present study, we assume that the same sample size (N = 6) of the aforementioned paper would be adequate to obtain a statistical power of 0.8 with an alpha level of 0.05.

As described in the previous paper, rats subjected to CCI of the infraorbital nerve were made allodynic and subsequently treated with a single intraperitoneal injection of the TRPA1 antagonist ADM_12 (30 mg/Kg) or its vehicle (saline) [15]. One hour after ADM_12/saline treatment, animals underwent the mechanical stimulation test, and subsequently, they were sacrificed according to the experimental protocol to which they were assigned (Supplementary Figure S3). For details related to the surgery procedure, the behavioral test, and treatment schedules, please refer to Demartini et al. [15].

### 4.2. Immunofluorescence Staining

Following transcardial perfusion with 4% paraformaldehyde, as previously described [15], the medullary segment containing the TNC was removed, post-fixed for 24 h in the same fixative, and subsequently transferred in solutions of sucrose at increasing concentrations (up to 30%) during the following 72 h. All samples were cut transversely to a thickness of 30 µm and placed on a freezing sliding microtome. Immunofluorescence staining of microglia and astroglia in the TNC region was performed as recently described [12]. Briefly, after phosphate-buffered saline rinses and blocking with normal goat serum, the sections were incubated overnight at 4 °C with a mouse anti-cluster of differentiation molecule 11b (anti-CD11b, 1:300, Serotec MCA275R, AbD Serotec, Oxfordshire, UK) primary antibody (to detect microglia) or rabbit antiglial fibrillary acidic protein (anti-GFAP, 1:500, Dako Z0334, Santa Clara, CA, USA) primary antibody (to label astrocytes). For the evaluation of TRPA1 expression in microglia and astroglia, the rabbit anti-TRPA1 (1:100, Immunological Sciences AB-84141, Roma, Italy) primary antibody was incubated with the mouse anti-CD11b (1:300, Serotec MCA275R, AbD Serotec, Oxfordshire, UK) or mouse anti-GFAP (1:1000, Immunological Sciences MAB-12029, Roma, Italy) primary antibodies. Goat anti-mouse AlexaFluor^®^ 488 or goat anti-rabbit AlexaFluor^®^ 594 (1:300, Life Sciences, A11001 and A11012, respectively, Thermo Fisher Scientific, Waltham, MA, USA) were used as secondary antibodies. Fluoroshield^TM^ with DAPI (Sigma-Aldrich F6057, St. Louis, MO, USA) was used to cover slices to label cell nuclei. Omission of the primary antibodies were performed in parallel as negative controls.

For each animal, four representative sections alongside the TNC were analyzed by an AxioSkop 2 microscope (Zeiss, Oberkochen, Germany) and a computerized image analysis system (AxioCam, Zeiss, Oberkochen, Germany), equipped with dedicated software (AxioVision Rel 4.2, Zeiss, Oberkochen, Germany). A researcher blind to the animal’s group assignment assessed the microglia and astroglia cell count by counting the CD11b- or GFAP-positive cells, respectively, from a stack of 16 pictures (1 μm thick, 20-fold magnification) of each section. The total numbers of CD11b- and GFAP-positive cells of the four sections were summed and expressed per mm^2^ [12]. In Supplementary Figure S4, the location of immunofluorescence images taken in the TNC area is reported.

### 4.3. Enzyme-Linked Immunosorbent Assay (ELISA)

Truncal blood (3 mL) was collected in BD Vacutainer^®^ K_3_EDTA Tubes and centrifuged at room temperature for 10 min at 1300 RCF for cytokine and HMGB1 plasma evaluations with commercial ELISA kits. TNF-alpha (cat: 865.000.096, Diaclone, Besançon, France), IL-1beta (cat: 670.040.096, Diaclone, Besançon, France), IL-6 (cat: 670.010.096, Diaclone, Besançon, France), IL-10 (cat: 670.070.096, Diaclone, Besançon, France), IL-4 (cat: 865.020.096, Diaclone, Besançon, France), and HMGB1 (E-EL-R0505, Elabscience Biotechnology Co., Houston, TX, USA) levels were measured using a CLARIOstar microplate reader (BMG LABTECH, Ortenberg, Germany).

### 4.4. Real-Time Polymerase Chain Reaction (rt-PCR)

The medulla (bregma: −13.30 to −14.60 mm) in toto and the upper CSC (C1-C2), as well as the TGs both ipsilateral and contralateral to the CCI or sham injury of the infraorbital nerve, were utilized for gene expression analysis. The inclusion of the medulla and the upper CSC in the evaluations was due to their anatomical relevance, as these regions encompass the TNC [63], receiving the nociceptive information from the trigeminal territory, including signals originating from the infraorbital nerve territory [64,65]. These samples were processed as previously described to evaluate the gene expression and microRNA levels, using rt-PCR (Table 1).

Total RNA was extracted using the Aurum Total RNA Fatty and Fibrous Tissue Module Kit (BIO-RAD, Hercules, CA, USA) in RNase-free conditions, and cDNA was generated with the iScript cDNA Synthesis Kit (BIO-RAD, Hercules, CA, USA). Fast Eva Green Supermix (BIO-RAD, Hercules, CA, USA) was used to evaluate gene expression. Glyceraldehyde 3-phosphate dehydrogenase (*GAPDH*), whose expression remains constant in all experimental groups, was used for normalization [15].

The total RNA extracted as described above was also used to evaluate miRNAs; cDNA was synthesized using MirXMirna First-Strand Synthesis (Takara-Diatech Labline, Jesi-Ancona, Italy), and miR-let-7b expression levels were determined using TB Green q-Rt PCR (Takara-Diatech, Labline, Jesi-Ancona, Italy). RNU6-6P (U6) was used as a housekeeping gene to standardize miRNA expression [66].

All samples were assayed in triplicate and averaged; the 2^−∆Ct^ method was used to evaluate the amount of mRNA normalized to *GAPDH* or *U6*.

### 4.5. Statistical Evaluation

Statistical analysis was performed using the GraphPad Prism program (version 8, GraphPad Software, San Diego, CA, USA). All data were tested for normality using the Kolmogorov–Smirnov normality test. Differences between groups were analyzed by the one-way or two-way analysis of variance (ANOVA) followed by Tukey’s multiple comparison test. A probability level of less than 5% was considered as significant.

## 5. Conclusions

The findings indicate that, beyond their established role in the nociceptive pathway, TRPA1 channels may also contribute directly or indirectly to pain-related inflammation, potentially through interactions with TLR4- and TLR7-mediated mechanisms at both the neuronal and glial levels.

This study lays the groundwork for future research to comprehensively define the interactions between TRPA1 channels and other critical components of inflammatory pathways involved in TN, potentially leading to the identification of dual therapeutic targets for chronic pain treatment.

## Figures and Tables

**Table 1:** Genes and primer sequences.

Gene	Forward Primer	Reverse Primer
*GAPDH*	AACCTGCCAAGTATGATGAC	GGAGTTGCTGTTGAAGTCA
*IL-10*	GCTCAGCACTGCTATGTTGC	CAGTAGATGCCGGGTGGTTC
*IL-4*	CAACAAGGAACACCACGGAG	TTTCAGTGTTGTGAGCGTGG
*GFAP*	GATGTAGGAGTGGGTAGGGC	CCCTCTCCGCATCCATACTT
*CD11b*	GAAGCCTTGGCGTGTGATAG	GAGCAGTTTGTTCCCAAGGG
*iNOS*	TGGCCTCCCTCTGGAAAGA	GGTGGTCCATGATGGTCACAT
*Arginase 1*	GACATCAACACTCCGCTGAC	TTGCCAATTCCCAGCTTGTC
*C3*	TCAGGGTCCCAGCTACTAGT	TGTTCAGATGTCCAGTGGCT
*S100a10*	TTCTATCACTAGTGGCGGGG	AAGGGTCCTGATCTGCTCAC
*HMGB1*	GGAACGGTTTGCCTTGCTTA	ACTTGACAGAGGCAGGATCC
*TLR4*	TATCGGTGGTCAGTGTGCTT	AGTCCTCATTCTGGCTCGAG
*TLR7*	CTGTCCTTGAGTGGCCTACA	TCAGATACCCAGGCATGTCC
*U6*	TGCGGGTGCTCGCTTCGGCAGC	CCAGTGCAGGGTCCGAGGT
*miR-let-7b*	CGGGGTGAGGTAGTAGGTTG	CAGGGAAGGCAGTAGGTTGT

*C3*: Complement 3; *CD11b*: cluster of differentiation molecule 11b; *GAPDH*: glyceraldehyde 3-phosphate dehydrogenase; *GFAP*: glial fibrillary acidic protein; *HMGB1*: high-mobility group box 1; *iNOS*: inducible nitric oxide synthase; *IL*: interleukin; *S100a10*: S100 Calcium-Binding Protein A10; *TLR*: Toll-like receptor; *U6*: RNU6-6P.

## Data Availability

Raw data are available upon reasonable request at www.zenodo.org (doi: 10.5281/zenodo.14941962).

## Supplementary Materials

The following supporting information can be downloaded at https://www.mdpi.com/article/10.3390/molecules30091884/s1, Figure S1: *IL-10* and *IL-4* mRNA levels; Figure S2. TRPA1 immunostaining; Figure S3. Experimental plan; Figure S4. Location of the immunofluorescence images in the trigeminal nucleus caudalis (TNC) area.

## Abbreviations

The following abbreviations are used in this manuscript:

ANOVA	analysis of variance
C3	complement 3
CCI	chronic constriction injury
CD11b	cluster of differentiation molecule 11b
CSC	cervical spinal cord
*GAPDH*	glyceraldehyde 3-phosphate dehydrogenase
GFAP	glial fibrillary acidic protein
IL	interleukin
iNOS	inducible nitric oxide synthase
ELISA	enzyme-linked immunosorbent assay
HMGB1	high-mobility group box 1
NF-Kb	nuclear factor kappa B
rt-PCR	real-time polymerase chain reaction
S100a10	S100 Calcium-Binding Protein A10
TG	trigeminal ganglion
TLR	Toll-like receptors
TN	trigeminal neuralgia
TNC	trigeminal nucleus caudalis
TNF-alpha	tumor necrosis factor-alpha
TRPA1	transient receptor potential ankyrin type-1
*U6*	RNU6-6P
