# Supplementary material for: Insights into the Involvement of TRPA1 Channels in the Neuro-Inflammatory Machinery of Trigeminal Neuralgia
Source: Molecules. 2025 Apr 23;30(9):1884. doi: 10.3390/molecules30091884 (PMC12073490; doi:10.3390/molecules30091884)
Supplement: Supplementary file 1 [file molecules-30-01884-s001.zip › Figure S1.pdf]

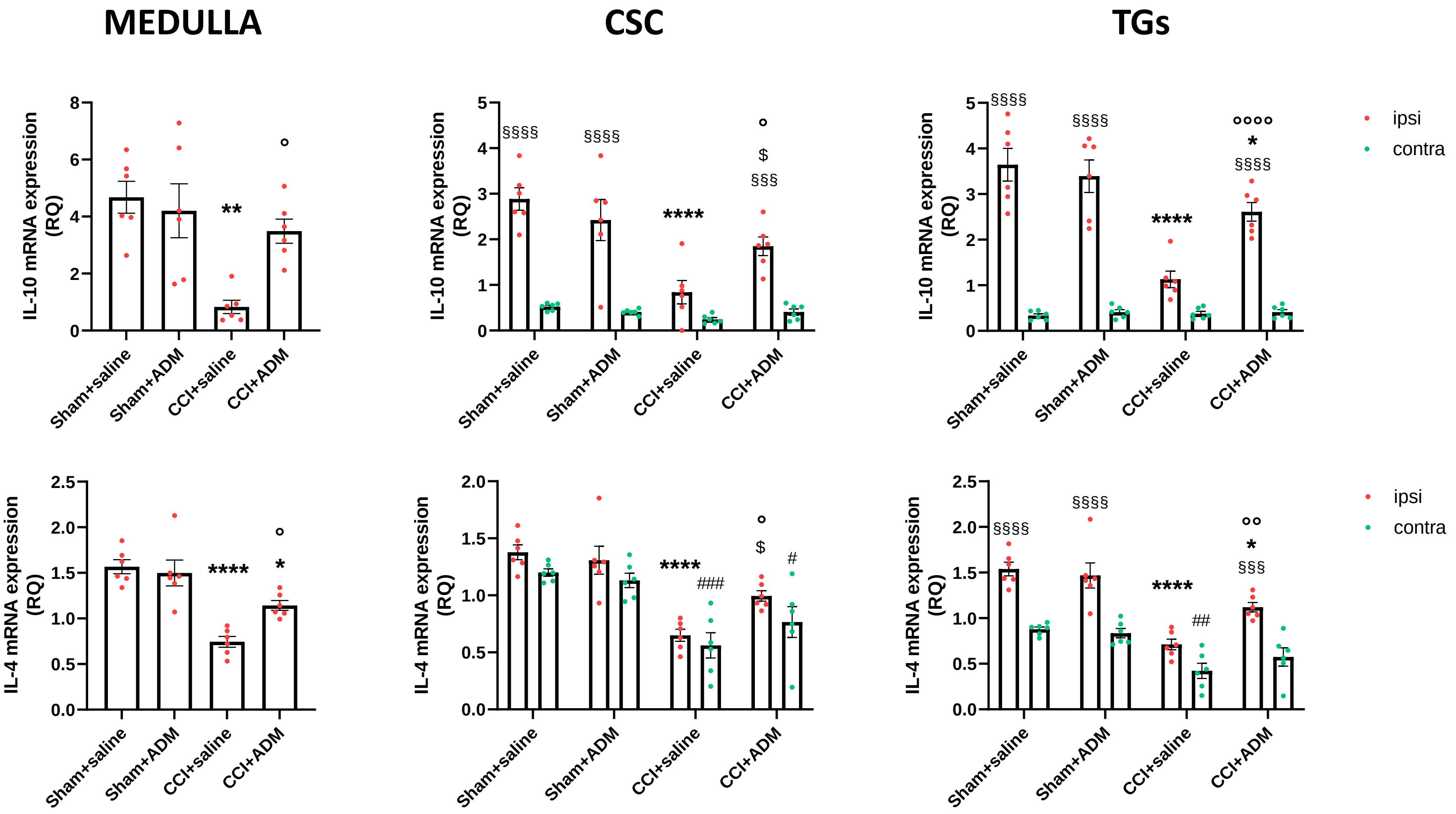

Figure S1. ***IL-10* and *IL-4* mRNA levels.** Gene expression levels (expressed as relative quantification, RQ) of *IL-10* and *IL-4* in medulla, cervical spinal cord (CSC) and trigeminal ganglia (TGs). Data are expressed as mean ± SEM. One way (medulla) and two way (CSC and TGs) analysis of variance (ANOVA) followed by Tukey's Multiple Comparison Test; *p<0.05, **p<0.01 and ****p<0.0001 vs. Sham+saline and Sham+ADM (ipsi); °p<0.05, °°p<0.01 and °°°°p<0.0001 vs. CCI+saline (ipsi); $p<0.05 vs. Sham+saline (ipsi); §p<0.05, §§§p<0.001 and §§§§p<0.0001 vs. contra; #p<0.05, ##p<0.01 and ###p<0.001 vs. Sham+saline and Sham+ADM (contra). N=6
