# Supplementary material for: Insights into the Involvement of TRPA1 Channels in the Neuro-Inflammatory Machinery of Trigeminal Neuralgia
Source: Molecules. 2025 Apr 23;30(9):1884. doi: 10.3390/molecules30091884 (PMC12073490; doi:10.3390/molecules30091884)
Supplement: Supplementary file 1 [file molecules-30-01884-s001.zip › Figure S2.pdf]

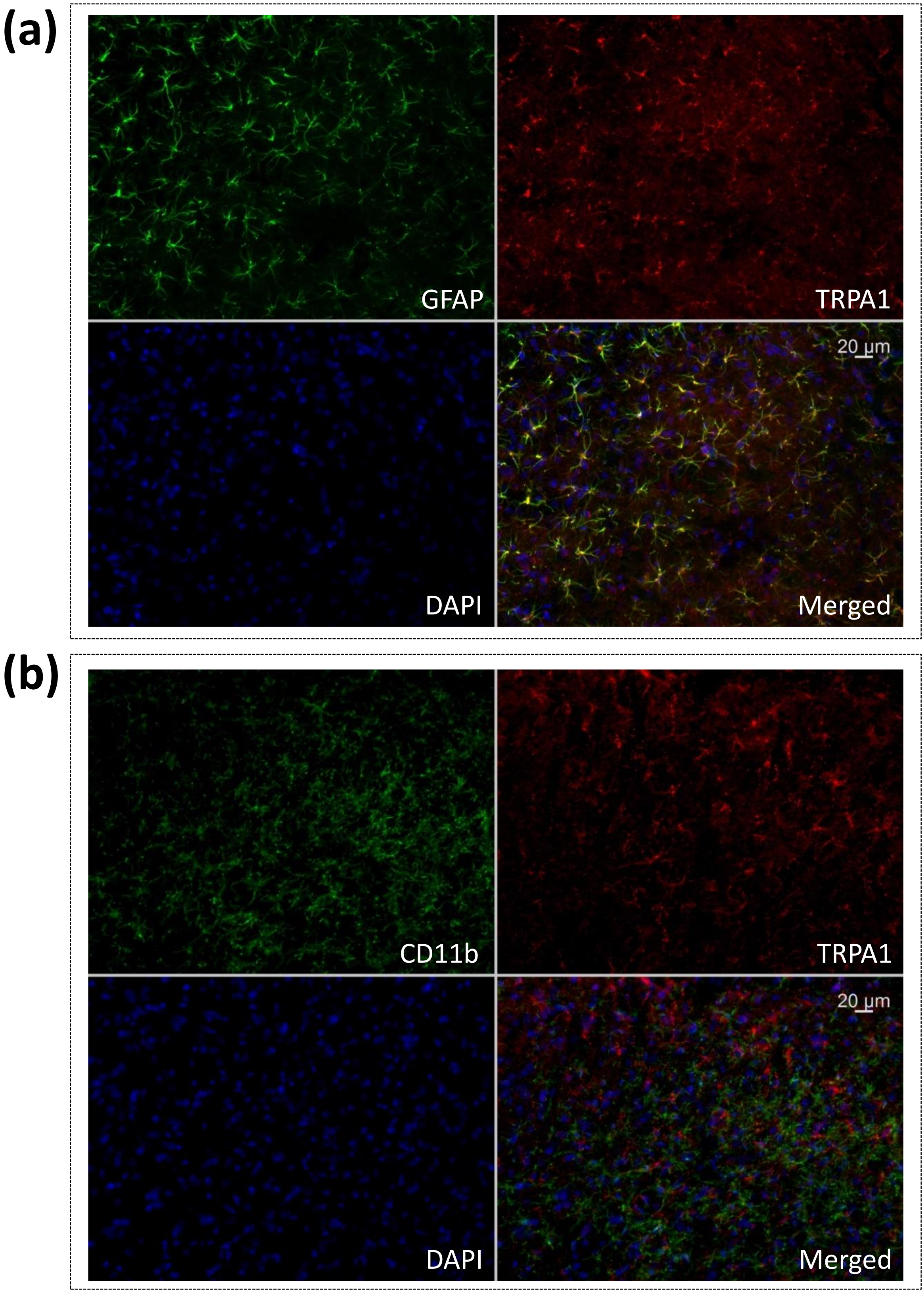

Figure S2. TRPA1 immunostaining. Representative photomicrographs (20x magnification) of TRPA1 (red) co-localization in GFAP-positive (green) astroglial cells (a) and CD11b-positive (green; 1:300, Serotec MCA275R) microglial cells (b) within the trigeminal nucleus caudalis. Cell nuclei stained with DAPI (blu). Scale bars: 20 µm.
