# Supplementary material for: Insights into the Involvement of TRPA1 Channels in the Neuro-Inflammatory Machinery of Trigeminal Neuralgia
Source: Molecules. 2025 Apr 23;30(9):1884. doi: 10.3390/molecules30091884 (PMC12073490; doi:10.3390/molecules30091884)
Supplement: Supplementary file 1 [file molecules-30-01884-s001.zip › Figure S3.pdf]

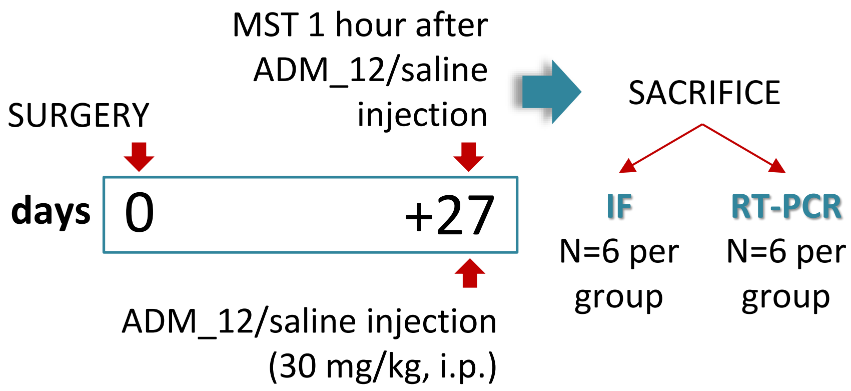

Figure S3. Experimental plan. Schematic representation of the experimental plan performed in the manuscript Demartini et al., 2018 (doi: 10.3390/ijms19113320).

Male Sprague Dawley rats were treated with ADM_12 or its vehicle (saline) 27 days after IoN-CCI/sham surgery. One hour after drug injection the animals underwent the mechanical stimulation test (MST) to evaluate trigeminal mechanical allodynia. At the end of the behavioral test rats were sacrificed and samples were collected according to the experimental protocol to which they were assigned: immunofluorescence (IF) or real-time PCR (RT-PCR).
