# Supplementary material for: Insights into the Involvement of TRPA1 Channels in the Neuro-Inflammatory Machinery of Trigeminal Neuralgia
Source: Molecules. 2025 Apr 23;30(9):1884. doi: 10.3390/molecules30091884 (PMC12073490; doi:10.3390/molecules30091884)
Supplement: Supplementary file 1 [file molecules-30-01884-s001.zip › Figure S4.pdf]

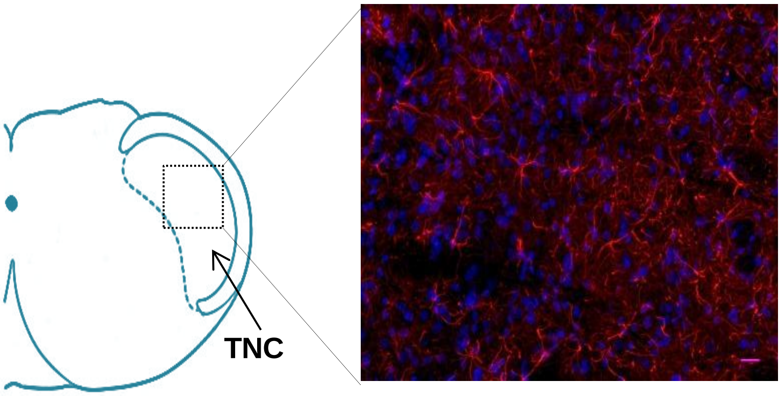

Figure S4. Location of the immunofluerescence images in the trigeminal nucleus caudalis (TNC) area. Site of acquisition of a representative photomicrograph at 20x magnification within the TNC. Red: GFAP staining of astroglia; Blue: cell nuclei stained with DAPI.
